# Salt Effects on the Mechanical Properties of Ionic Conductive Polymer: A Molecular Dynamics Study

**DOI:** 10.1021/acsmaterialsau.3c00098

**Published:** 2024-02-01

**Authors:** Harish Gudla, Kristina Edström, Chao Zhang

**Affiliations:** Department of ChemistryÅngström Laboratory, Uppsala University, Lägerhyddsvägen 1, Box 538, 75121 Uppsala, Sweden

**Keywords:** Solid-State Batteries, Polymer Binder, Mechanical Properties, Self-Healing, Electrolyte, Molecular Simulation

## Abstract

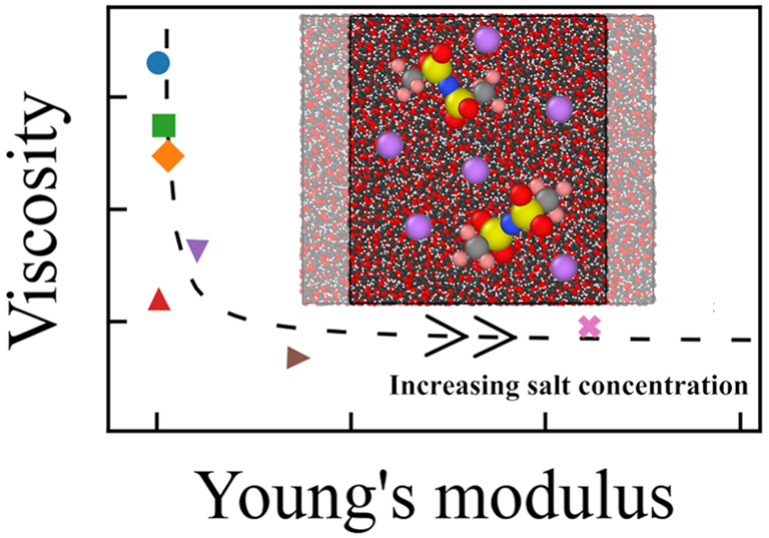

Functional polymers can be used as electrolyte and binder materials in solid-state batteries. This often requires performance targets in terms of both the transport and mechanical properties. In this work, a model ionic conductive polymer system, i.e., poly(ethylene oxide)-LiTFSI, was used to study the impact of salt concentrations on mechanical properties, including different types of elastic moduli and the viscoelasticity with both nonequilibrium and equilibrium molecular dynamics simulations. We found an encouragingly good agreement between experiments and simulations regarding Young’s modulus, bulk modulus, and viscosity. In addition, we identified an intermediate salt concentration at which the system shows high ionic conductivity, high Young’s modulus, and short elastic restoration time. Therefore, this study laid the groundwork for investigating ionic conductive polymer binders with self-healing functionality from molecular dynamics simulations.

## Introduction

Electrochemical energy storage, in particular, batteries, is a key enabler for the green energy transition and the deployment of electric vehicles. This has led to ever-increasing activities from both academia and industry with focuses on discovering new battery materials and cell chemistry leading to much higher energy density. However, due to the complexity of novel materials, they can be difficult to implement in battery products at scale. To address this issue, large-scale research initiatives, e.g., the BATTERY 2030+,^1^ have identified thematic areas, e.g. integrating smart functionalities of sensing and self-healing,^2^ into to the battery design.

One of the promising approach to implement the self-healing functionality into the next generation anode materials (Si as a prominent example) is to explore functional polymers as binder materials.^3−5^ For example, functional groups that involve hydrogen bonding^6−8^ is a popular choice for polymer binders that can mitigate the large volume expansion of Si anodes during the cycling. Other types of self-healing mechanisms,^9,10^ such as dynamical covalent bonds,^11^ ionic cross-linking^12^ and host–guest interactions,^13^ are also interesting options.

In all of these cases, an understanding of the mechanical properties of the ionic conductive polymer is necessary. Besides its electrochemical stability, a good ionic conductive polymer should satisfy the requirement of both ionic conductivity (≥10^–5^ S cm^–1^ at 25 °C) and mechanical strength (≥30 MPa at 25 °C).^14,15^ In this regard, molecular modeling^16^ can be rather useful to disentangle different factors that influence the mechanical properties of ionic conductive polymers and to extract design principles.

In contrast to ionic transport properties (e.g., transference number) where much has been understood recently with the help of molecular modeling,^17−26^ the mechanical properties of ionic conductive polymers are less studied,^27−31^ in particular, at atomistic scale. Therefore, in this work, we used a model ionic conductive polymer system, i.e., poly(ethylene oxide)-lithium bis(trifluoromethane)sulfonimide (PEO-LiTFSI), and all-atom molecular dynamics (MD) simulations to study the impact of salt concentrations on mechanical properties, including different types of elastic moduli and the viscoelasticity. It is found that all-atom force fields popularly used in the studies of ion transport in polymer systems can reproduce quite well the experimental results of Young’s modulus, bulk modulus, and viscosity. Despite the general trade-off between the transport property and the mechanical property as bounded by the Maxwell relation, we are able to identify an intermediate salt concentration at which the system possesses both high ionic conductivity and high Young’s modulus. Regarding the self-healing capability, we show that the elastic restoration time is correlated with the Young’s modulus in a nonlinear manner, which is interesting for further investigation.

In the following, we first present the theory of elasticity and viscoelasticity as well as the nonequilibrium and equilibrium MD methods used to investigate these mechanical properties. It follows with the results of salt effects on both elastic moduli and the relaxation modulus. Then, we present our attempt to identify an optimal salt concentration and quantify the self-healing capability. At last, a conclusion of this study and a perspective for future works are also provided.

## Theory and Methods

### Elastic Moduli from Nonequilibrium MD

According to Hooke’s law for elasticity,^32^ the 6 × 6 elastic constant matrix C is determined by the partial derivatives of the stress tensor, σ_*ij*_, with respect to the deformation or strain ε_*kl*_ is,
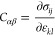
1where {*i*,*j*,*k*,*l*} ∈{*x*,*y*,*z*} and {α, β} ∈{1,2,3} . With the Voigt notation *xx* → 1, *yy* → 2, *zz* → 3, *yz* → 4, *xz* → 5 and *xy* → 6, the stiffness matrix ***C*** involving 21 unique elements can be written as follows
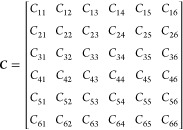
For an isotropic and cubic system, ***C*** is only dependent on two variable λ and μ called Lamé’s constants,^33^
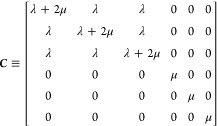
A least-squares procedure can be used to obtain Lamé’s constants according to eqs 2–5,^33^
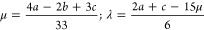
2
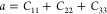
3
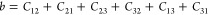
4
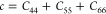
5Further, they can be used to calculate elastic moduli such as Young’s modulus (*E*), shear modulus (*G*), bulk modulus (*B*), and Poisson’s ratio (ν) according to the following equation.
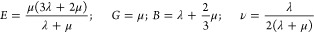
6

**Figure 1:**
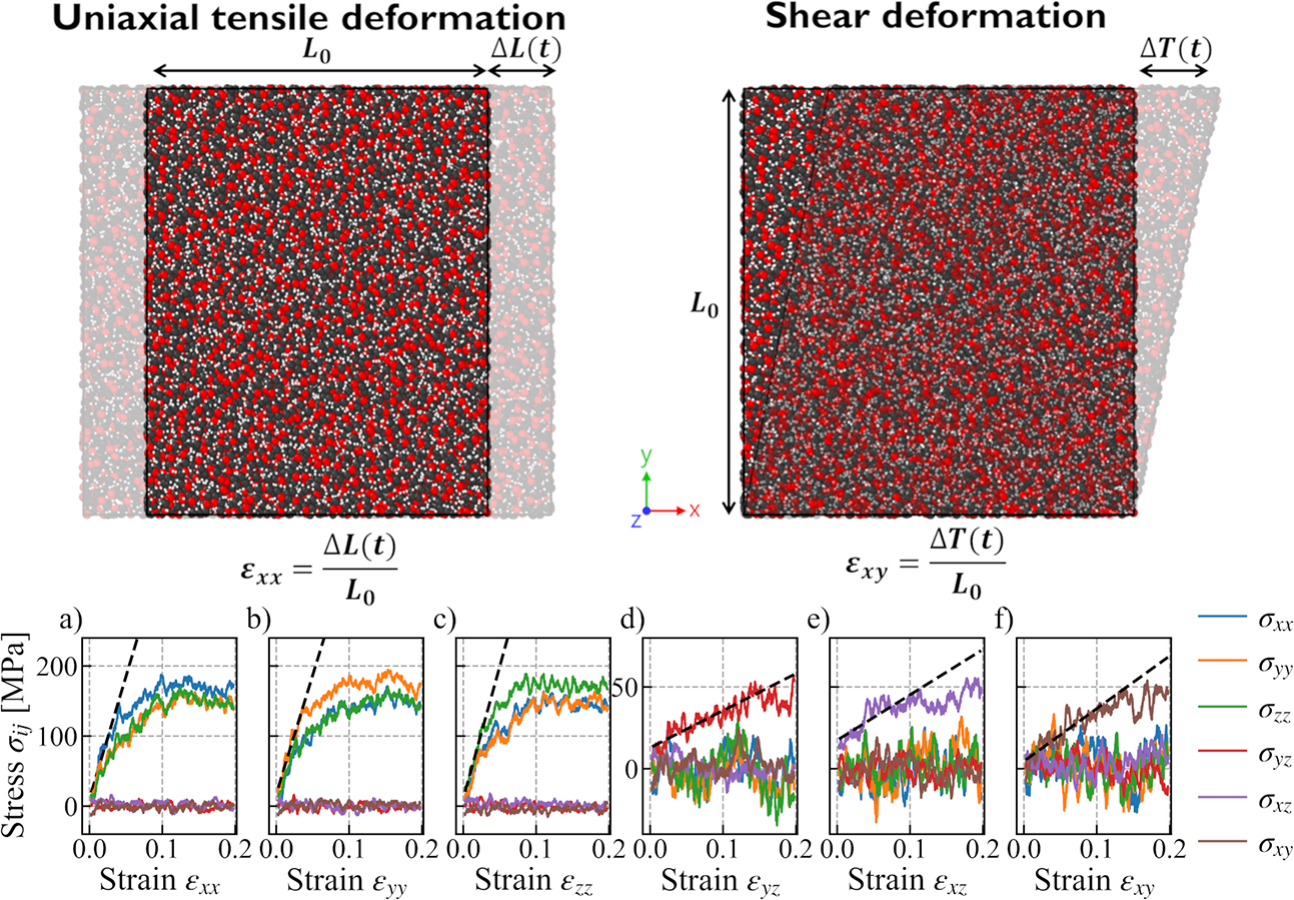
(top) Initial and final configurations of uniaxial tensile deformation and shear deformation simulations; (bottom a-f) six stress tensors plotted against strain in six nonequilibrium deformation simulations for the neat PEO system at a strain rate ε̇ of 5 × 10^9^ s^–1^ and *T*–*T*_g_ ≈ 120 K. Black dashed lines are linear fits to obtain *C*_*αβ*_ values.

A series of deformations, i.e. uniaxial tensile deformation and shear deformation (see Figure 1 top), were applied to the periodic simulation cell in order to estimate values of matrix element *C*_*αβ*_.^34^ For the uniaxial tensile deformation simulation, a strain was applied to the *x* direction and the remaining two dimensions (*yz*) were unchanged. This was repeated for the *y* and *z* directions, while keeping the remaining (*xz* and *xy*, respectively) dimensions fixed. Likewise, for three shear deformation simulations where shear strains were applied to *yz*, *xz* and *xy* planes. All deformations were in the positive direction, the strain was applied in a continuous fashion at every time step at a constant rate and six different strain rates varying from 10^8^ - 5 × 10^10^*s*^–1^ were considered in each case. For example, as shown in Figure 1 bottom, in the case of the deformation of the *x* direction, the σ_*xx*_ was plotted as a function of the strain ε_*xx*_ up to 20%, and the slope of this curve in the elastic regime over 5% of strain was obtained. Similarly, plots of σ_*yy*_ and σ_*zz*_ versus strain ε_*xx*_ and the corresponding slopes were obtained. These results were placed in the first column of the **C** matrix. The first three columns of the **C** matrix were therefore obtained from the three independent tensile deformation simulations in *x*, *y* and *z* directions. Instead, the last three columns were obtained from the three independent shear deformation simulations in *yz*, *xz*, and *xy* planes.

### Viscoelastic Properties from Equilibrium MD

From the Green–Kubo relation, the relaxation modulus *G*(*t*) of the system can be obtained from the autocorrelation functions of off-diagonal stress component ⟨σ_*xy*_(*t*)σ_*xy*_(0)⟩recorded during the equilibrium MD simulations.^35^ In the isotropic systems, *G*(*t*) can be obtained by averaging autocorrelations over the symmetrized traceless stress tensor (τ_*ij*_) components according to eq 7 to reduce the statistical error.^36^
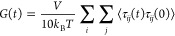
7
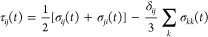
8where *V* is the volume of the system, *T* is the temperature and *k*_B_ is the Boltzmann constant.

The storage modulus *G*′(ω) and the loss modulus *G*″(ω) can be computed from the in-phase (real) and out-of-phase (imaginary) components of the relaxation modulus in the frequency domain (eq 9).
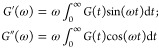
9To reduce the fluctuations in the stress time-autocorrelation functions, the multitau correlator method^37^ was used to calculate *G*(*t*) by implementing the python code “multipletau”.^38^ The numerical evaluations of *G*′(ω) and *G*″(ω) were carried out by following the method proposed by Adeyemi *et*. *al*.^39,40^

Then, the modulus of the frequency-dependent viscosity |η*(ω)|can then be estimated from the storage and loss moduli by using the following expression:
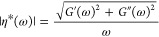
10

Taking the zero frequency limit, one obtains equilibrium viscosity η:
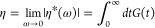
11

Equivalently, the equilibrium viscosity can also be obtained from the following expression:
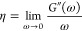
12

### MD Simulations of PEO-LiTFSI Systems

The GAFF force field parameters^41^ and simulation protocol for PEO-LiTFSI systems (25 monomer units in each PEO chain with the molecular weight of 1.11 kg/mol) at six different concentrations *c* [Li/EO] (the ratio of Li to ether oxygen) can be found in our previous studies.^22,23,25,26^ In this study, all MD simulation were carried out using LAMMPS^42^ instead of GROMACS^43^ for the convenience of computing mechanical properties. To ensure the consistency with our previous studies, glass transition temperatures and Nernst–Einstein ionic conductivity (σ_NE_) at different concentrations were computed using two codes but the same force field parameters and compared (see Figure S1 in the Supporting Information). Nonequilibrium MD simulations were performed for different simulation times depending on the strain rate (see Table 2 in the Supporting Information), and equilibrium MD simulations were carried out for 500–600 ns (see Table 1 in the Supporting Information). For nonequilibrium MD simulations, we have applied a Nosé–Hoover thermostat^44^ with SLLOD equations of motion^45^ at *T* – *T*_g_ ≈ 60 K (room temperature) and *T* – *T*_g_ ≈ 120 K (about 430 K) respectively. For equilibrium MD simulations, Nosé–Hoover thermostat^44^ and barostat^46^ were applied at *T* – *T*_g_ ≈ 120 K and 1 bar.

## Results and Discussion

### Effects of Strain Rate and Salt Concentration on Elastic Properties

**Figure 2:**
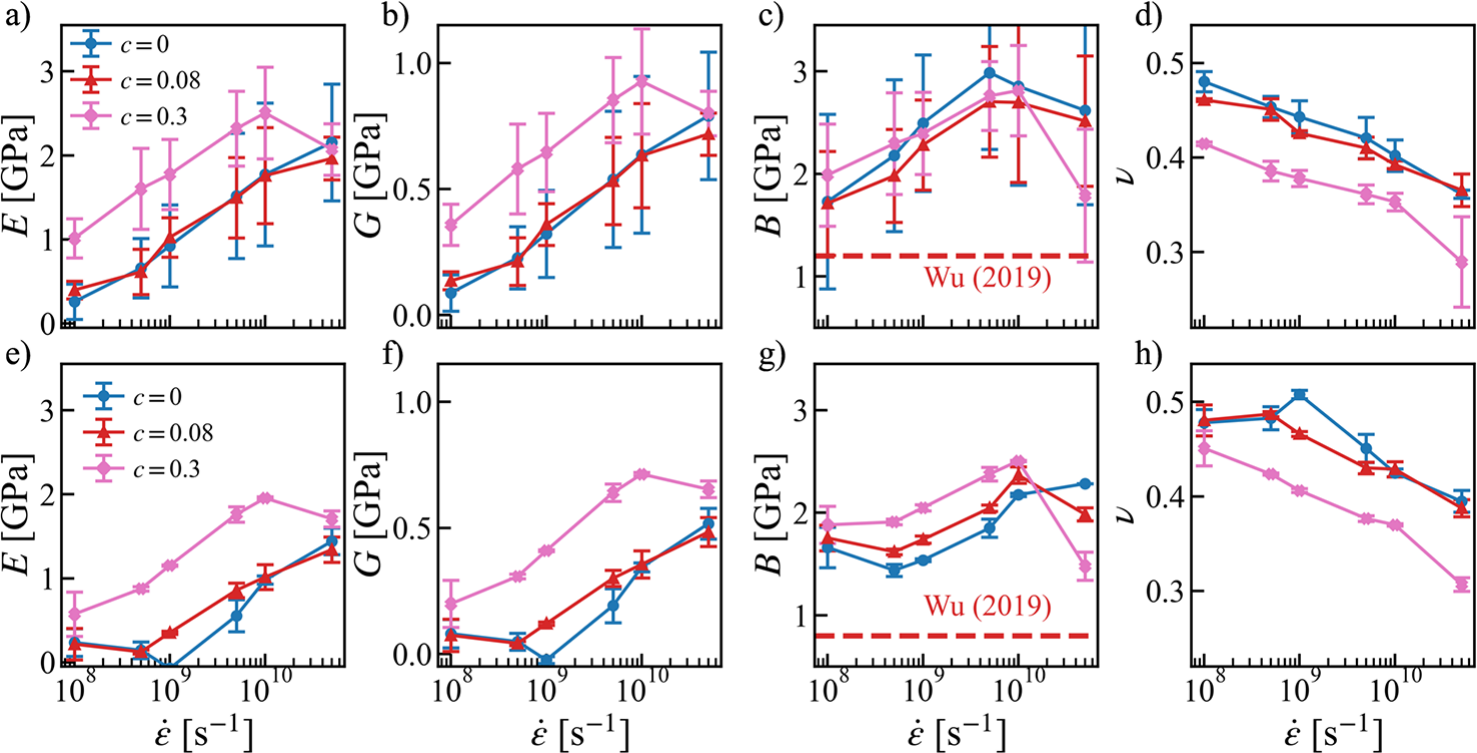
(a,e) Young’s modulus *E*; (b,f) shear modulus *G*; (c,g) bulk modulus *B*; and (d,h) Poisson’s ratio ν as a function of strain rate (ε̇) for the neat PEO and the PEO-LiTFSI systems at two different concentrations (*c* [Li/EO] = 0.02, 0.3) and at *T* – *T*_g_ ≈ 60 K (a–d) and 120 K (e–h). Red dashed line: The experimental bulk modulus for the neat PEO system at the respective temperatures from ref (47).

The elastic moduli and Poisson’s ratio calculated from eq 6 as a function of strain rate at two different effective temperatures (*T* – *T*_g_) and three salt concentrations (*c*) are shown in Figure 2. The error bars were estimated using the standard deviation from simulations with 5 different initial configurations. As shown in Figure 2, both Young’s modulus *E* and shear modulus *G* were found to increase with the strain rate, as the polymer chains have less time to respond to the strain, which makes the chains look stiffer. The opposite was seen for the Poisson’s ratio, which approaches a perfect incompressible rubber state at a lower strain rate.^48^ In contrast, bulk modulus *B* seems to be insensitive to the strain rate. This explains why the simulation results agree quite well with the experimental bulk modulus,^47^ despite of the order of magnitude difference in the strain rate. Since the Lamé constant λ is the dominating contribution to *B* (see eq 6, Figure S3b,d), this also explains why the Poisson’s ratio has an opposite strain-rate dependence as compared to *E* and *G*.

**Figure 3:**
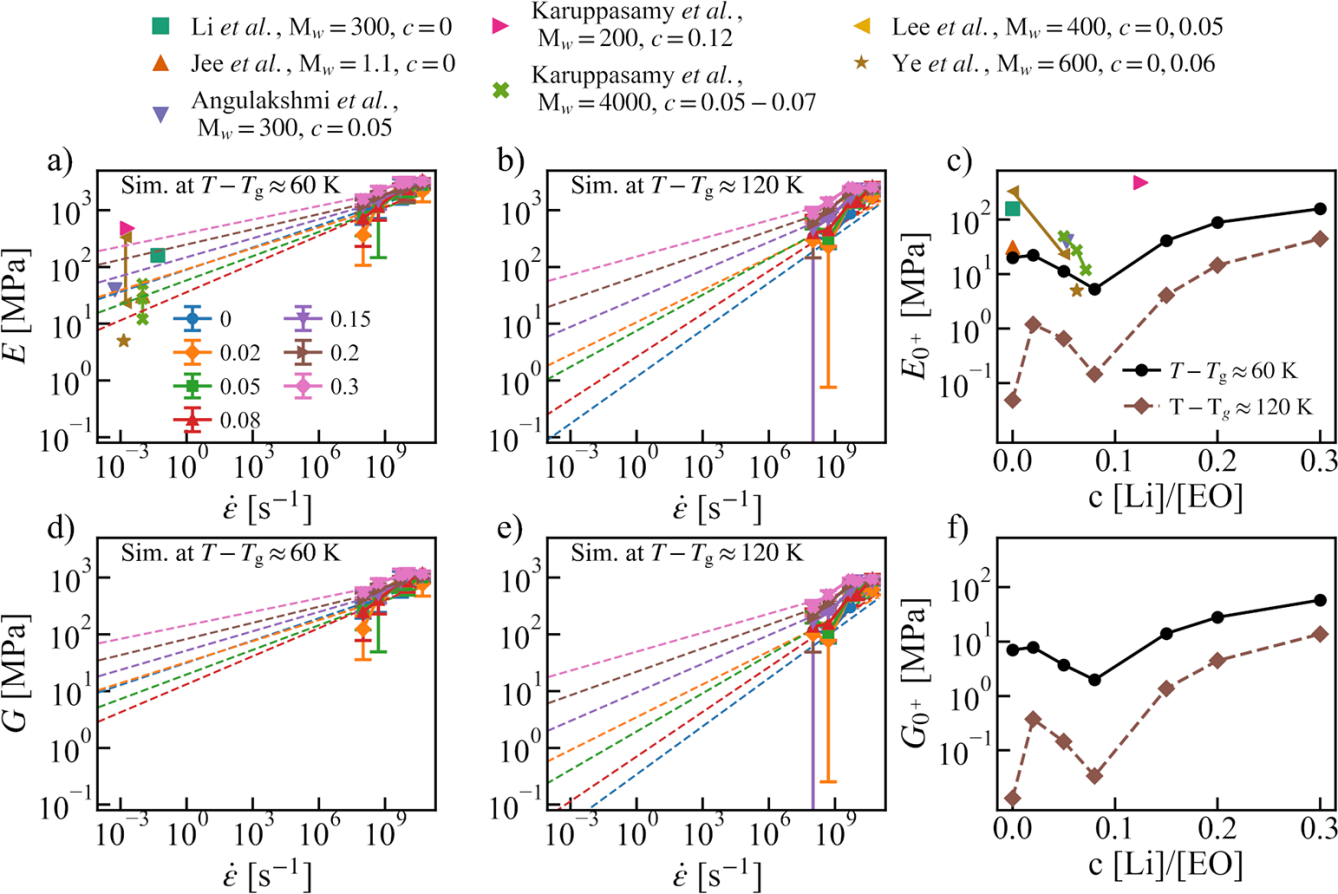
(a,b) Young’s modulus *E* and (d,e) shear modulus *G* as a function of strain rate (ε̇) for the neat PEO and the PEO-LiTFSI systems at temperatures *T* – *T*_g_ ≈ 60 and 120 K from nonequilibrium MD simulations. (c) near zero strain-rate Young’s modulus *E*_0^+^_ and (f) near zero strain-rate shear modulus *G*_0^+^_ as a function of salt concentration. Dashed lines are linear fits to calculate Young’s modulus at near zero strain rate *E*_0^+^_. The experimental Young’s modulus for PEO based electrolytes at room temperature^49−55^ were also plotted for comparison and summarized in the Supporting Information. The concentration *c* [Li/EO] is the ratio of Li to ether oxygen.

The strain rates used in the simulations are 8–10 orders of magnitude higher in comparison to the experimental strain rates (10^–4^–10^–2^ s^–1^). Therefore, in order to compare with experimental Young’s modulus, the extrapolation was used. In Figure 3a,b,d, and e, the *E* and *G* were plotted as a function of strain rates and linear fittings in the log–log scale were carried out to estimate the near zero strain-rate Young’s modulus *E*_0^+^_ i.e., *E* at a strain rate (10^–2^ s^–1^) similar to the experimental one. As shown Figure 3a, the extrapolations (dashed line) to the low strain rate are in very good agreement with the experimental values for PEO-based electrolytes in the same effective temperatures *T*–*T*_g_ ≈ 60K.^49−55^ As shown in Figures 2 and 3b,e, it is clear that increasing the effective temperature will reduce both the Young’s modulus and the shear modulus.

The salt concentration has a nonmonotonic effect on both elastic moduli and Poisson’s ratio, which can be already seen in Figure 2. At high salt concentration *c* = 0.3, both *E* and *G* become much larger compared to the neat PEO system. However, at low and moderate concentrations, the effect can be the opposite and the increase in temperature may further convolute the situation. This can be clearly seen in Figures 3c and 3f, which shows the salt concentration-dependence of *E*_0^+^_ and *G*_0^+^_. Simulations results agree with the experimental trend that the elastic moduli decrease with a moderate increment in salt concentration (Figures 3c). However, a further increase in the salt concentration beyond *c* = 0.08 leads to enhanced elastic moduli instead, as predicted by simulations.

### Salt Effects on Relaxation Modulus and Viscosity

**Figure 4:**
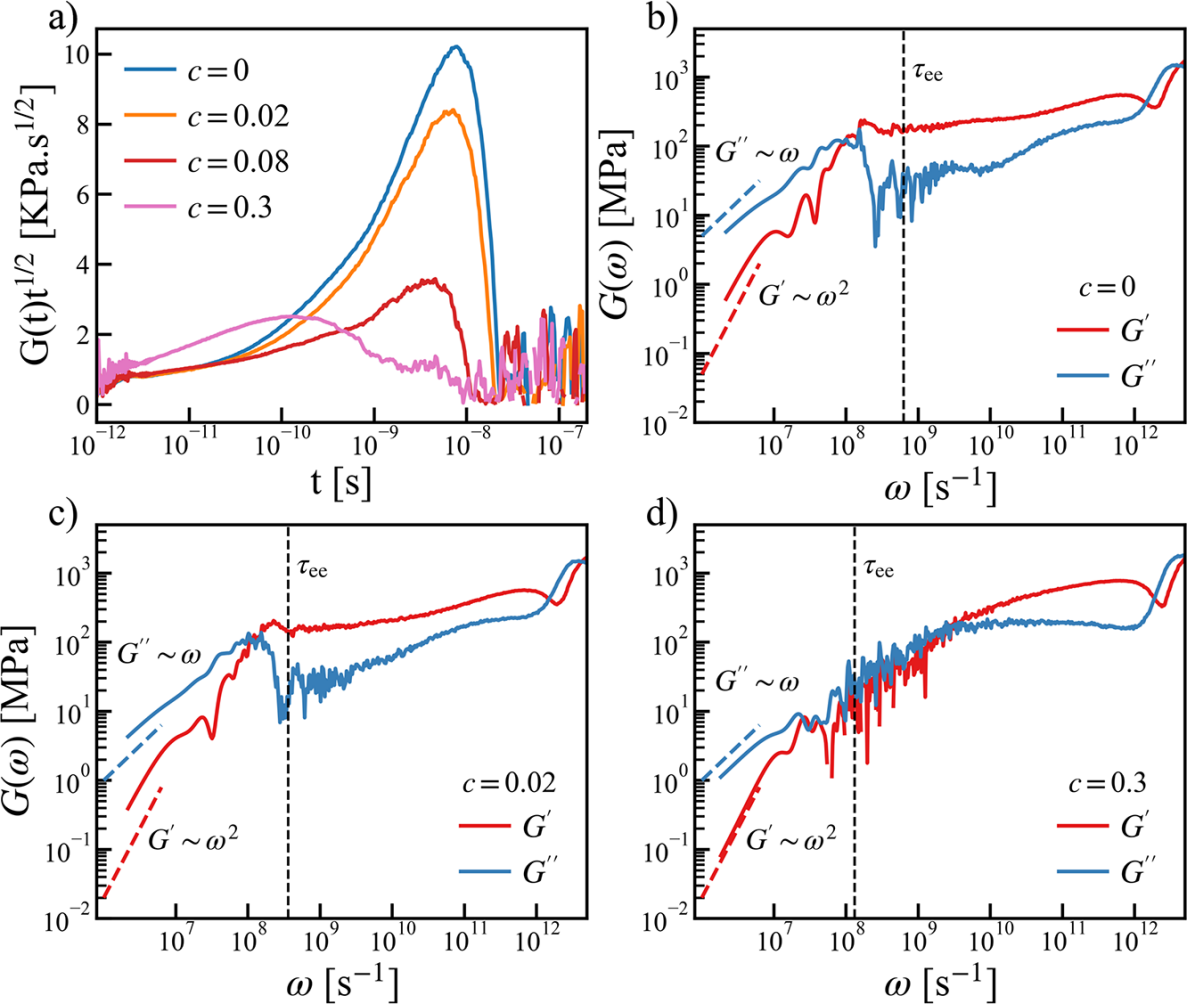
(a) Relaxation modulus *G*(*t*) scaled with *t*^1/2^ for PEO and PEO-LiTFSI at different concentrations (*c* = 0.02, 0.08, 0.3). (b–d) Storage modulus *G*′(ω) and loss modulus *G*″(ω) for the neat PEO and PEO-LiTFSI systems at *c* = 0.02, 0.3 at *T* – *T*_g_ ≈ 120 K. The blue and red dashed lines correspond to the relations *G*″ ∼ ω and *G*′ ∼ ω^2^. The vertical dashed lines indicate the end-to-end relaxation time τ_*ee*_ (see Supporting Information).

The shear stress relaxation moduli *G*(*t*) calculated from EMD simulations for different salt concentrations are shown in Figure 4a. Likhtman et al.^56^ has identified four time scales to the stress relaxation modulus by using a simple bead–spring model of polymer melt, namely, (i) the oscillatory behaviors at short time arises due to bond length relaxations; (ii) the colloidal or glassy mode due to collisions between atoms; (iii) the Rouse dynamics i.e. polymer relaxation according to the Rouse theory *G*(*t*)∼ *t*^–1/2^; (iv) the polymer entanglement. Given the rigid bond model and low molecular weight systems used in this study, it is natural for us to focus on identifying the Rouse dynamics. If the system follows the Rouse dynamics, then the product *G*(*t*)*t*^1/2^ would be equal to a constant. As shown in Figure 4a, with the addition of salt, these dynamics seem to gradually deviate from the Rouse dynamics. The system with the highest salt concentration (*c* = 0.3) shows the largest deviation from Rouse theory. It is also interesting to note that the large peak shown in Figure 4a resembles the entanglement behavior of high molecular weight systems revealed in ref (56), and we will come back to this point in the paragraph below.

The storage modulus *G*′(ω) and the loss modulus *G*″(ω) for the neat PEO (*c* = 0) and two concentrations (*c* = 0.02 and *c* = 0.3) were plotted in Figure 4b–d. In all cases, a clear crossover from the solid-like behaviors *G*′(ω) > *G*″(ω) to the liquid-like behaviors *G*″(ω) > *G*′(ω) was observed. In the low frequency range, the asymptotic behaviors of viscoelastic liquid^59^*G*″ ∼ ω and *G*′ ∼ ω^2^ were also evinced in our simulations. In addition, a second crossover at high frequency were seen in all cases from simulations. It is worth noting that the linear rheology experiments are usually conducted at much lower frequency (longer time scale) and at higher molecular weights^58,60^ and the second crossover between *G*′ and *G*″ signals the entangled polymer dynamics.^61^ Nevertheless, similar observations made here suggest that the viscoelastic properties from a low molecular weight system and equilibrium MD simulations may emulate the realistic polymer dynamics at much higher molecular weight and longer time scale.

**Figure 5:**
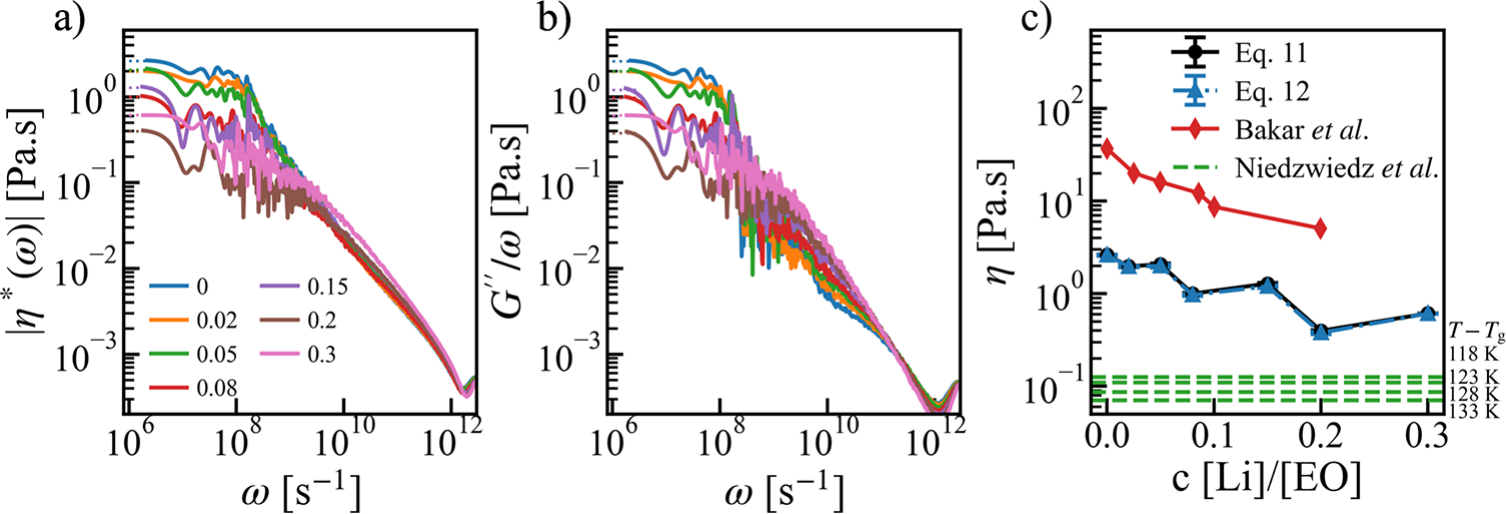
(a) Modulus of the frequency-dependent viscosity |η*(ω)|and (b) *G*″(ω) /ω for the neat PEO and PEO-LiTFSI systems at different concentrations and at temperature *T* – *T*_*g*_ ≈ 120 K. (b) Comparing the equilibrium viscosity η computed from eqs 11 and 12 with experimental values^57,58^ for PEO-LiTFSI systems as a function of salt concentration.

To make a further connection to the experiment, we plotted the modulus of the complex viscosity in Figure 5a for different salt concentrations at *T* – *T*_g_ = 120 *K*. As expected from eq 11, we observed that at lower frequency, the values tend to become a constant and can be used to estimate the equilibrium viscosity η. The same applies to the estimator based on eq 12 using the loss modulus *G*″ (see Figure 5b). The results of η are shown in Figure 5c. With an increase in concentration, η tends to decrease with oscillations. A similar trend was also observed in the experiments^57^ for PEO-LiTFSI systems (the red line in Figure 5c). However, the viscosity values reported in the experiment are 1 order of magnitude higher than the simulation results obtained here because of the difference in the molecular weight (20 kg/mol in experiment versus 1.1 kg/mol in simulation). Indeed, our results come closer to experimental reference^58^ measured at similar molecular weight (green dashed lines in Figure 5c).

Before closing this section, it is worth noting that both the storage and loss modulus decrease with the increment in salt concentration (see Figure 4b–d), which is similar to that of the equilibrium viscosity η. This may appear in contradiction with the finding shown in Figure 3f that the shear modulus *G*_0^+^_ increases with salt doping, especially at high concentration. However, in the solid-like regime, i.e., *G*′(ω) > *G*″(ω) at high frequency, the magnitude of *G*′ indeed becomes larger by adding salts. Therefore, this contrast just reflects the opposite effects of salts on the shear modulus of ionic conductive polymers at different time scales. Furthermore, according to the Maxwell model, which is a combination of a Hookean solid and a Newtonian fluid, the shear stress relaxation time is determined by the ratio η/*G*_0^+^_. This means the shear stress relaxation time should decrease more rapidly with the increase of the salt concentration. Indeed, this is borne, as shown by the crossover time *G*′ = *G*″ at the low frequency in Figure 4.

### Optimal Salt Concentration and Self-Healing Capability

As stated in the Introduction, searching ionic conductive polymers that satisfy the requirements for both transport and mechanical properties and demonstrate self-healing functionality is an emerging topic in the battery field. Therefore, it would be interesting to address this point from our simulations.

**Figure 6:**
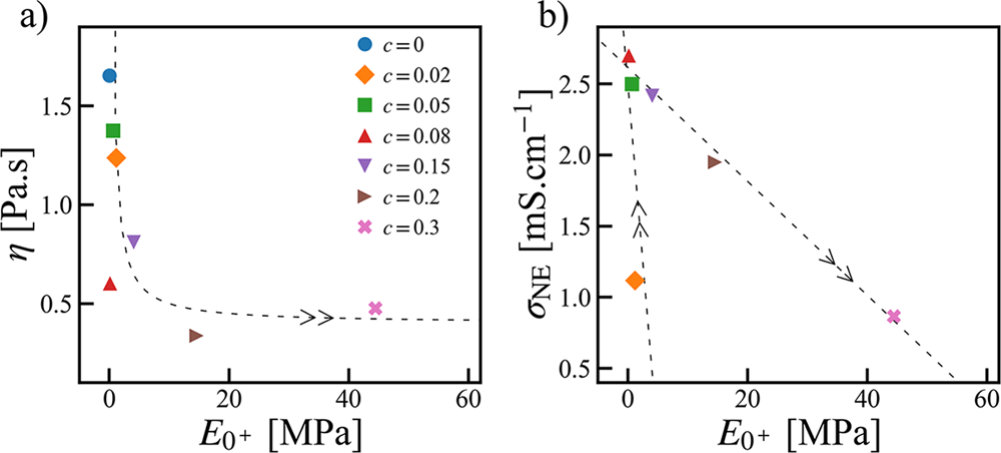
Correlations between the Young’s modulus at near zero strain rate *E*_0^+^_ with (a) the equilibrium viscosity η and (b) the Nernst–Einstein conductivity σ_NE_ at *T* – *T*_g_ ≈ 120 K. The dashed lines are guides to the eye. The arrow heads indicate the direction in which the salt concentration increases.

As shown in Figure 6a, the salt effects on the equilibrium viscosity η and the near zero strain-rate Young’s modulus *E*_0^+^_ are opposite, which makes these two quantities anticorrelated to each other. This means, there is a trade off between a good Newtonian fluid and a good Hookean solid, which is what the Maxwell relation implies.^62^

However, when making the correlation between the ionic conductivity σ_NE_ and the Youngs’ modulus *E*_0^+^_, the situation is more interesting. Despite that σ_NE_ and *E*_0^+^_ are also anticorrelated in general, there exist two regimes. From the low to intermediate concentrations, σ_NE_ goes up rapidly while *E*_0^+^_ slightly goes down; with a further increment in the concentration, σ_NE_ goes down while *E*_0^+^_ goes up in a comparable degree. As a consequence, there is an intermediate salt concentration at *c* [Li/EO] = 0.2 where the system processes both high ionic conductivity and high Young’s modulus. It is worth noting that at a lower temperature (*T* – *T*_g_ = 60 K), σ_NE_ goes down (σ_NE_ = 5.3 × 10^–5^ S.cm^–1^) while *E*_0^+^_ goes up (*E*_0^+^_ = 89 MPa). Therefore, an optimal salt concentration may be located where the requirements for both ionic conductivity and mechanical strength as mentioned in the Introduction can be satisfied.

**Figure 7:**
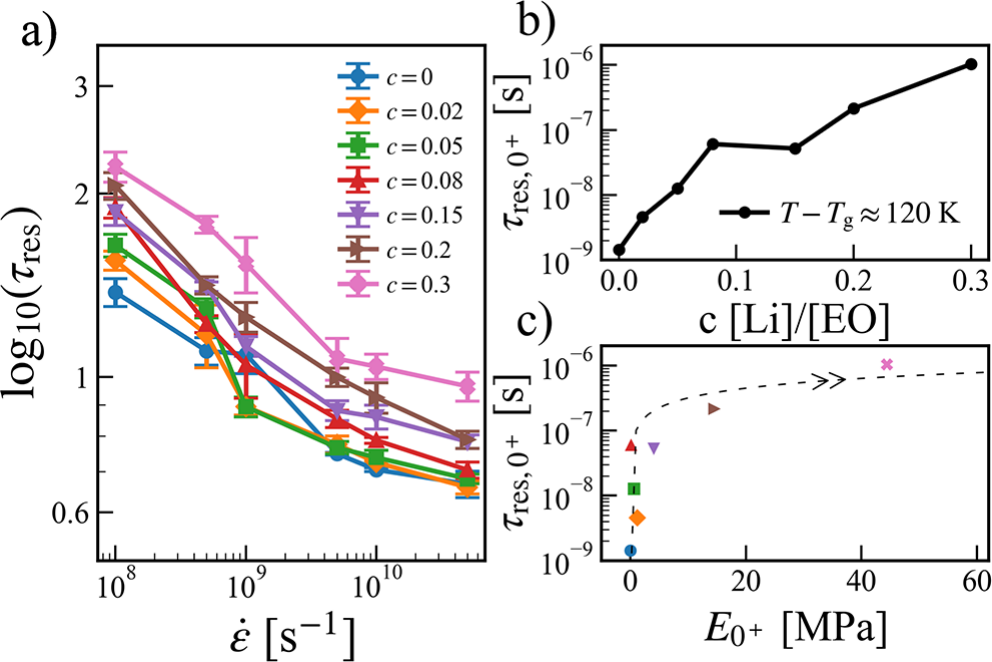
(a) Elastic restoration time τ_res_ as a function of strain rate ε̇ at different salt concentrations. (b) Elastic restoration time at the near zero strain rate τ_res, 0^+^_ as a function of salt concentration. (c) Correlations between the elastic restoration time at the near zero strain rate τ_res, 0^+^_ and the near zero-strain rate Young’s modulus *E*_0^+^_ at *T* – *T*_g_ ≈ 120 K. The dashed line is a guide for the eye, and the arrow heads indicate the direction where the salt concentration increases.

The final point that we want to address here is about the self-healing capability of ionic conductive polymers. Here, we define the self-healing capability as the elastic restoration time τ_res_ for the system to restore its equilibrium density after expansion under a tensile strain (see the Supporting Information). This is the self-healing process prior to the mechanical damage for tearing the polymer apart and creating an interface. As shown in Figure 7a, despite all simulations used to compute the restoration time τ_res_ started with the same expansion rate of 20%, the resulting τ_res_ depends on the strain rate used in the generated these initial structures. This suggests that the self-healing capability depends on the history of how fast the deformation has taken place. Therefore, we used the elastic restoration time τ_res, 0^+^_ extrapolated to the near zero strain rate, as a benchmark index.

As shown in Figure 7b, τ_res,0^+^_ increases with increasing salt concentration. This implies that τ_res, 0^+^_ correlate positively with the Young’s modulus *E*_0^+^_, as seen in Figure 7c. In other words, ionic conductive polymers of the highest Young’s modulus do not have the best self-healing capacity. However, this correlation is nonlinear, and the system with *c* = 0.2 shows a good balance between high Young’s modulus and short elastic restoration time.

## Conclusions

In this study, we have carried out all-atom MD simulations to investigate salt effects on the mechanical properties of poly(ethylene oxide)-LiTFSI as a model ionic conductive polymer system with both nonequilibrium and equilibrium methods. The focus has been on both the elastic moduli and the relaxation modulus.

Regarding the elastic moduli, it is found that all-atom force fields commonly used in studying ion transport can reproduce quite well the experimental results of Young’s modulus and bulk modulus. Further, we found that the Poisson’s ratio goes down by increasing the strain-rate while the opposite happens to the Young’s modulus *E* and shear modulus *G*. We confirmed the experimental observation that in the low concentration regime, the Young’s modulus becomes smaller by adding salts. However, our simulation also revealed that a further increase of the salt concentration can enhance Young’s modulus instead.

In terms of the relaxation modulus, our MD simulations showed that the low molecular weight system and equilibrium MD simulations may emulate the entanglement features of the relaxation modulus, which should only happen in principle to polymer systems at much higher molecular weight and longer time scale. Moreover, the computed viscosity η is in good agreement with experimental results at a comparable molecular weight, and we confirmed the experimental observation of a decrement in viscosity with salt concentration. The same trend was also seen for both the storage modulus *G*′ and the loss modulus *G*″ at the low frequency regime from simulations.

Besides comparing the results with experiments and studying the trends, we were able to identify an intermediate salt concentration *c* [Li/EO] = 0.2 at which the system possesses both high ionic conductivity and high Young’s modulus. This intermediate salt concentration also leads to a short elastic restoration time, which can be relevant to the self-healing capacity of ionic conductive polymer.

We expect that more follow-up studies will come out to relate the self-healing capacity of ionic conductive polymers to their mechanical properties with all-atom MD simulations. In particular, questions such as how to define self-healing capacities from MD simulations and how to relate them to measurable experimental quantities should be addressed. By making these efforts, we would be able to understand the molecular mechanisms of self-healing functionality and extract design principles for novel polymer binder materials.

## Supporting Information Available

Further simulation details; comparison between LAMMPS and GROMACS simulations with the same force field implementation; strain-rate dependence of Lamé’s constants μ and λ; summary of experimental references on Young’s modulus and viscosity; calculations of elastic restoration time τ_res_ (PDF)
