# Supplementary material for: Salt Effects on the Mechanical Properties of Ionic Conductive Polymer: A Molecular Dynamics Study
Source: ACS Mater Au. 2024 Feb 1;4(3):300–7. doi: 10.1021/acsmaterialsau.3c00098 (PMC11083113; doi:10.1021/acsmaterialsau.3c00098)
Supplement: Supplementary file 1 — mg3c00098_si_001.pdf [file mg3c00098_si_001.pdf]

Supporting Information: Salt effects on the mechanical properties of ionic conductive polymer: a molecular dynamics study

Harish Gudla, Kristina Edström, and Chao Zhang*

Department of Chemistry—Ångström Laboratory, Uppsala University, Lägerhyddsvägen 1, Box 538, 75121 Uppsala, Sweden

E-mail: chao.zhang@kemi.uu.se

Further simulation details

The MD simulation boxes for bulk PEO and PEO:LiTFSI at different salt concentrations were obtained by adding 0, 100, 250, 400, 750, 1000 and 1500 LiTFSI molecules to 200 polymer chains with 25 monomer EO units, corresponding to a [Li]/[EO] concentration ratio of 0, 0.02, 0.05, 0.08, 0.15, 0.20, and 0.3, respectively. All MD simulations were run with a time step of 1 fs and the thermostat and barostat coupling constants were set to 0.1 and 2.0 ps, respectively. The SHAKE constraint algorithm[1] was used to constrain the H-atoms in the polymer chains with an accurate tolerance of 10^4 for every 20 fs. The simulation time for equilibrium MD simulations for NPT equilibration and production runs is given in Table 1. The stress tensors were recorded every 5 fs and the trajectory is saved every 5 ps which is used for calculating diffusion coefficients. The traceless stress tensors were then calculated for the whole simulation using the saved stress tensors and to calculate $G(t)$, time-autocorrelation functions were calculated using multipletau python code.[2]

Table 1: The simulation time in ns for NPT equilibration and production runs for PEO and PEO:LiTFSI systems at different concnetrations.

System	c	Equilibration run [ns]	Production run [ns]
PEO	0	10	550
PEO:LiTFSI	0.02	10	544
	0.05	10	525
	0.08	10	604
	0.15	10	606
	0.2	10	573
	0.3	10	576

The non-equilibrium MD simulations i.e. tensile and shear deformation simulations were carried out at six different strain rates and to reach a deformation of 20% different simulation times were required as given in Table 2. The stress tensors were recorded for every 10 fs and different averaging times were considered to have a uniform statistics in different strain

rates, as given in Table 2.

Table 2: The simulation times and averaging times in ps at different strain rates for non-equilibrium MD simulations.

Strain rate [s^{-1}]	Simulation time [ps]	Averaging time [ps]
5×10^{10}	4	0.01
10^{10}	20	0.05
5×10^{9}	40	0.1
10^{9}	200	0.5
5×10^{8}	400	1
10^{8}	2000	5

Comparison between LAMMPS and GROMACS simulations with the same force field implementation

The procedure of computing T_g and the Nernst-Einstein conductivity σ_{NE} can be found in previous publications.[3,4]

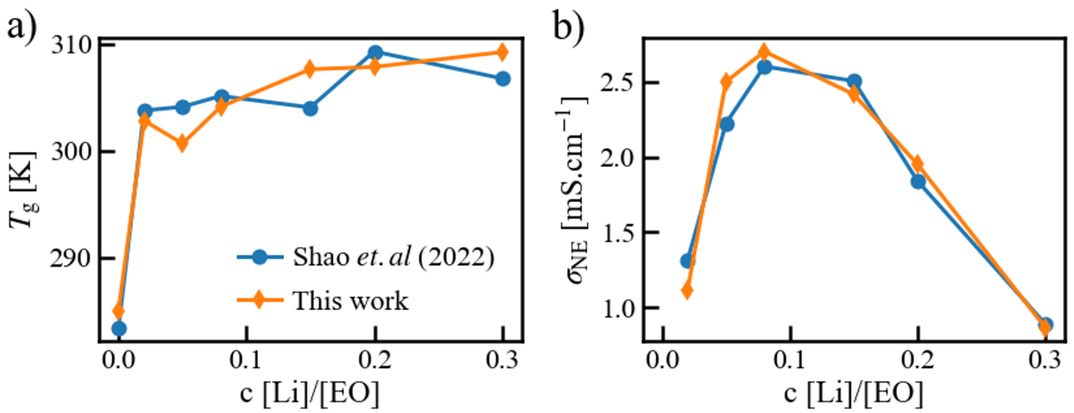

Figure S1: Comparing a) the glass transition temperature T_g and b) the Nernst-Einstein ionic conductivity σ_{NE} from GROMACS and LAMMPS simulations with the same GAFF parameterization.

Calculation of end-to-end relaxation time

The end-to-end distance autocorrelation function $C(t)$ of polymer chains for all systems were plotted in Fig. S2, the shaded region corresponds to standard deviation from three different time origins. The relaxation time τ_{ee} is obtained by fitting $C(t)$ to a simple exponential decay function according to Eq.1.

$$C(t) = \frac{\langle R_{ee}(t_0) \cdot R_{ee}(t_0 + t) \rangle}{R_{ee}^2(t_0)} = \exp\left(\frac{-t}{\tau_{ee}}\right) \tag{1}$$

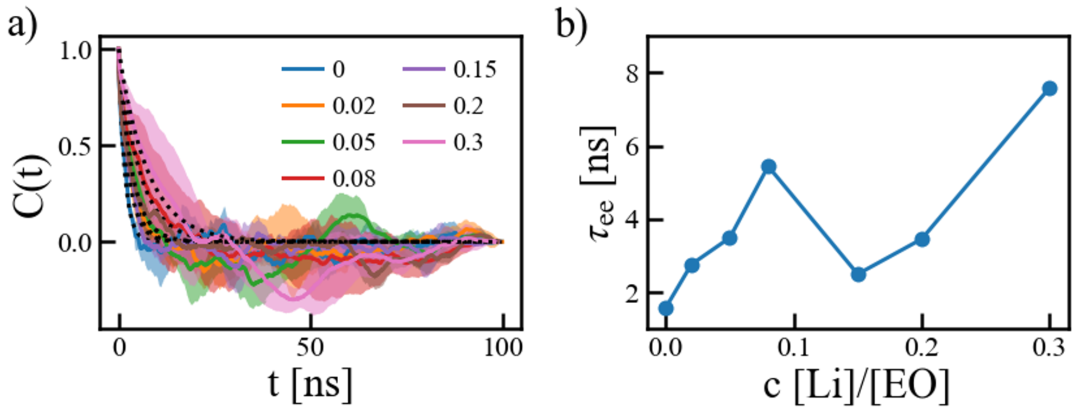

Figure S2: a) Autocorrelation functions of end-to-end distance vector for PEO and PEO-LiTFSI systems at different salt concentrations. Dashed lines are fits to the exponential decay functions. b) End-to-end relaxation time τ_{ee} as a function of salt concentration.

Strain-rate dependence of Lamé's constants μ and λ

The Lamé's constants, μ and λ calculated for all the systems according to the Eq. 2 in main text at different temperatures, were plotted in Fig. S3. Since, Young's modulus E and shear modulus G are directly proportional to μ, the similar strain rate dependence can also been observed there. From Figs. S3b,d, the λ values are independent of the strain rates, which was also reflected from bulk modulus B. Finally, the Poisson's ratio ν was inversely dependent on μ, therefore, an opposite strain-rate dependence was observed.

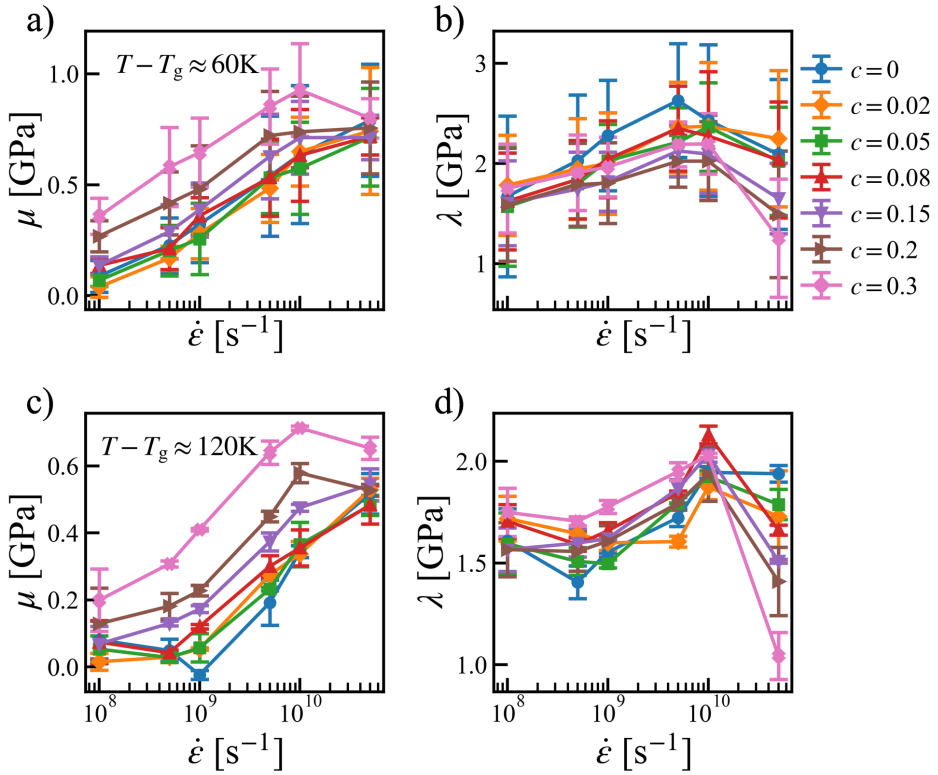

Figure S3: Lamé's constants, μ (a,c) and λ (b,d) as function of strain rates at different concentrations and temperatures $T - T_{\mathrm{g}} \approx 60$K and $T - T_{\mathrm{g}} \approx 120$K.

Summary of experimental references on the Youngs' modulus and viscosity

To compare the Young's modulus and equilibrium viscosities obtained from our simulations with the experimental data, a number of references were found from the literature for the PEO-based electrolytes systems and their details can be found in Table 3. In Refs. 5–8, the Young's modulus was estimated from the stress-strain curves in the elastic regime, i.e. 2-5% of strain.

Table 3: Experimental data extracted from literature. The name of the reference (Ref.), the polymer electrolyte system (System) and glass transition temperature T_g,K in parentheses, polymer molecular weight (M_w) in kg.mol^{-1}, salt concentration c [Li]/[EO], strain rate $\dot{\varepsilon}$ in s^{-1}, measured temperature T in K or RT for room temperature, Young's modulus E in MPa and measured equilibrium viscosity η in Pa·s.

Ref.	System	M_w	c	$\dot{\varepsilon}$	T	E	η
Li *et al.*[5]	PEO	300	-	0.05	RT	160	-
Jee *et al.*[9]	PEO	1.1	-	0.01	RT	31	-
Angulakshmi *et al.*[6]	PEO:LiTFSI	300	0.05	0.00055	RT	40	-
Karuppasamy *et al.*[7]	PEO:LiTFSI	200	0.125	0.002	RT	480	-
Karuppasamy *et al.*[10]	PEO:LiBNFSI	4000	0.05	0.01	RT	500	-
			0.0625			27.5	
			0.071			12	
Lee *et al.*[11]	PEO:LiTFSI	400	0	0.017	RT	332.5	-
			0.05			23.2	
Ye *et al.*[8]	PEO:$LiClO_4$	600	0.0625	0.0014	RT	5	-
Bakar *etal.*[12]	PEO:LiTFSI (T_g ≈230)	20	0	-	348	-	36875
			0.025				20129
			0.05				16150
			0.085				12241
			0.1				8682
			0.2				5075
Niedzwiedz *et al.*[13]	PEO (T_g =190)	0.89	-	-	308	-	0.126
					313		0.110
					318		0.087
					323		0.071

Calculations of elastic restoration time τ_{res}

The final configurations from the uniaxial tensile deformed system (20% deformation) in all three directions and six strain rates were used in the NPT simulations with Nosé-Hoover thermostat[14] and barostat[15] for 5 ns and densities were saved every 0.05 ps. The choice of simulation time is sufficient as the densities reach the equilibrium values within this time scale. The ratio of equilibrium density ($\rho_{\text{eq.}}$) with instantaneous density (ρ) at different strain rates and salt concentrations were plotted in Fig. S4 and were then fitted with a simple exponential decay function to calculate elastic restoration time τ_{res}.

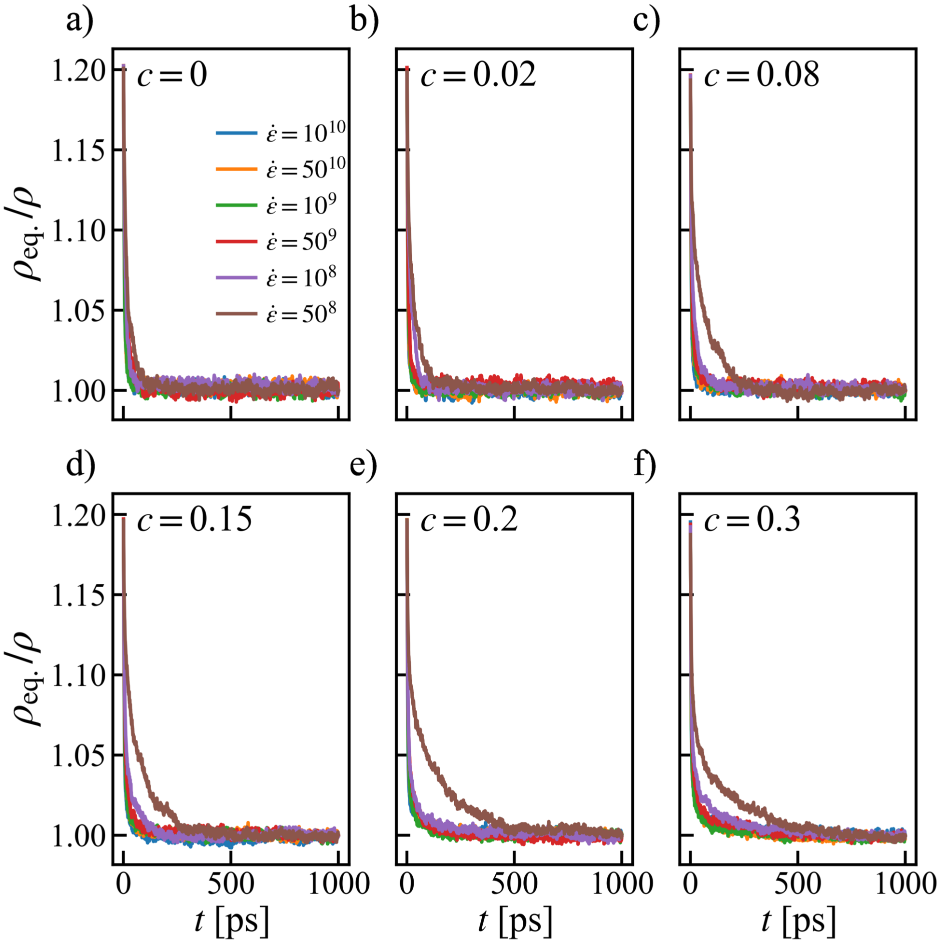

Figure S4: The ratio of equilibrium density $\rho_{eq.}$ with instantaneous density ρ at different strain rates for the neat PEO (a) and PEO-LiTFSI systems at different salt concentrations (b to f).

References

(1) Ryckaert, J. P.; Ciccotti, G.; Berendsen, H. J. Numerical integration of the cartesian equations of motion of a system with constraints: molecular dynamics of n-alkanes. *Journal of Computational Physics* **1977**, *23*, 327–341.

(2) Paul Müller, Python multiple-tau algorithm (Version 0.3.3). `https://pypi.python.org/pypi/multipletau/`, 2012; Accessed: 2023-09-01.

(3) Gudla, H.; Zhang, C.; Brandell, D. Effects of Solvent Polarity on Li-Ion Diffusion in

Polymer Electrolytes: An All-atom Molecular Dynamics Study with Charge Scaling. *J. Phys. Chem. B* **2020**, *124*, 8124–8131.

(4) Shao, Y.; Gudla, H.; Brandell, D.; Zhang, C. Transference Number in Polymer Electrolytes: Mind the Reference-Frame Gap. *J. Am. Chem. Soc.* **2022**, *144*, 7583–7587.

(5) Li, J.; Zhu, K.; Yao, Z.; Qian, G.; Zhang, J.; Yan, K.; Wang, J. A promising composite solid electrolyte incorporating LLZO into PEO/PVDF matrix for all-solid-state lithium-ion batteries. *Ionics* **2020**, *26*, 1101–1108.

(6) Angulakshmi, N.; Kumar, R. S.; Kulandainathan, M. A.; Stephan, A. M. Composite Polymer Electrolytes Encompassing Metal Organic Frame Works: A New Strategy for All-Solid-State Lithium Batteries. *J. Phys. Chem. C* **2014**, *118*, 24240–24247.

(7) Karuppasamy, K.; Rhee, H. W.; Reddy, P. A.; Gupta, D.; Mitu, L.; Polu, A. R.; Sahaya Shajan, X. Ionic liquid incorporated nanocomposite polymer electrolytes for rechargeable lithium ion battery: A way to achieve improved electrochemical and interfacial properties. *J. Ind. Eng. Chem.* **2016**, *40*, 168–176.

(8) Ye, Y.-S.; Wang, H.; Bi, S.-G.; Xue, Y.; Xue, Z.-G.; Zhou, X.-P.; Xie, X.-L.; Mai, Y.-W. High performance composite polymer electrolytes using polymeric ionic liquid-functionalized graphene molecular brushes. *J. Mater. Chem. A* **2015**, *3*, 18064–18073.

(9) Jee, A.-Y.; Lee, H.; Lee, Y.; Lee, M. Determination of the elastic modulus of poly(ethylene oxide) using a photoisomerizing dye. *Chem. Phys.* **2013**, *422*, 246–250.

(10) Karuppasamy, K.; Kim, D.; Kang, Y. H.; Prasanna, K.; Rhee, H. W. Improved electrochemical, mechanical and transport properties of novel lithium bisnonafluoro-1-butanesulfonimidate (LiBNFSI) based solid polymer electrolytes for rechargeable lithium ion batteries. *J. Ind. Eng. Chem.* **2017**, *52*, 224–234.

(11) Lee, J.; Howell, T.; Rottmayer, M.; Boeckl, J.; Huang, H. Free-Standing PEO/LiTFSI/LAGP Composite Electrolyte Membranes for Applications to Flexible Solid-State Lithium-Based Batteries. *J. Electrochem. Soc.* **2019**, *166*, A416–A422.

(12) Bakar, R.; Darvishi, S.; Aydemir, U.; Yahsi, U.; Tav, C.; Menceloglu, Y. Z.; Senses, E. Decoding Polymer Architecture Effect on Ion Clustering, Chain Dynamics, and Ionic Conductivity in Polymer Electrolytes. *ACS Appl. Energy Mater.* **2023**, *6*, 4053–4064.

(13) Niedzwiedz, K.; Wischnewski, A.; Pyckhout-Hintzen, W.; Allgaier, J.; Richter, D.; Faraone, A. Chain dynamics and viscoelastic properties of poly(ethylene oxide). *Macromolecules* **2008**, *41*, 4866–4872.

(14) Evans, D. J.; Holian, B. L. The Nose–Hoover thermostat. *J. Chem. Phys.* **1985**, *83*, 4069–4074.

(15) Nosé, S.; Klein, M. Constant pressure molecular dynamics for molecular systems. *Mol. Phys.* **1983**, *50*, 1055–1076.
